# Evaluation of Renal Gene Expression of Protein Kinase C (PKC) Isoforms in Diabetic and Nondiabetic Proliferative Glomerular Diseases

**DOI:** 10.1100/tsw.2008.108

**Published:** 2008-08-31

**Authors:** Salwa Ibrahim, Laila Rashed, Sawsan Fadda

**Affiliations:** ^1^ Department of Internal Medicine, Cairo University, Egypt; ^2^ Department of Medical Biochemistry, Cairo University, Egypt; ^3^ Department of Pathology, Cairo University, Egypt

**Keywords:** protein kinase C (PKC), diabetic nephropathy, lupus nephritis, mesangioproliferative glomerulonephritis

## Abstract

The protein kinase C (PKC) family consists of 13 members categorized as conventional or novel depending on whether diacylglycerol, calcium, or phosphatidylserine is required for activation. High glucose leads to activation of different forms of PKC across tissue types, thus determining the kind of diabetes-induced organ damage. PKC β was reported to have a positive role in B-lymphocyte activity through activation of NF-κB, leading to various immune disorders. We examined renal expression of two PKC isoforms α and β in renal biopsies of patients with diabetic nephropathy, lupus nephritis (LN) (Class 3-4), and mesangioproliferative glomerulonephritis (MPGN) to explore the role of each isoform in different glomerular diseases. PKC α and β gene expression was studied by quantitative real-time reverse transcription-PCR in 20 patients with type 2 diabetes and proteinuria (serum creatinine 2.04 ± 0.85 mg/dl, 24-h urinary protein 3.61 ± 1.75 g, eGFR 37.85 ± 17.89 ml/min/1.73 m^2^), 20 patients with proliferative LN (serum creatinine 1.67 ± 1.50 mg/dl, 24-h urinary protein 4.46 ± 5.01 g, eGFR 69.62 ± 40.93 ml/min/1.73 m^2^), and 20 patients with MPGN (serum creatinine 3.32 ± 2.79 mg/dl, 24-h urinary protein 4.65 ± 4.11 g, eGFR 32.62 ± 29.56 ml/min/1.73 m^2^). Normal tissues from the normal pole of four kidneys removed because of renal tumor served as controls. PKC α gene expression was significantly increased in diabetic kidneys compared to LN and MPGN (316.95 ± 152.94 µg/ml vs. 185.97 ± 32.13 and 195.46 ± 46.45 µg/ml, *p* < 0.05). PKC β gene expression was significantly increased in the LN and MPGN groups compared to the diabetic nephropathy group (41.01 ± 14.03 and 39.93 ± 16.41 µg/ml, respectively, vs. 18.20 ± 4.91 µg/ml, *p* < 0.05). Significant correlation was noted between the PKC α gene concentrations and proteinuria in diabetic patients. Renal expression of PKC α and β genes in control tissues were significantly lower compared to diabetic kidneys, LN, and MPGN groups (32.31 ± 0.36 and 4.67 ± 2.41 µg/ml, respectively, *p* < 0.001). The study revealed enhanced renal gene expression of both PKC isoforms α and β in diabetic kidney tissues, LN, and MPGN, but in different patterns. PKC α gene expression was significantly increased in diabetic patients with chronic kidney disease. The increased expression of the PKC β gene in LN and MPGN highlights its role in regulation of the immune system. This may represent potential therapeutic targets for prevention of progressive kidney injury in diabetic and proliferative glomerular diseases.

## INTRODUCTION

Diabetic nephropathy is a leading cause of end-stage renal disease (ESRD), accounting for 35% of new cases requiring dialysis therapy in the western world and Asian countries[1]. Hyperglycemia causes glomerular dysfunction through activation of the protein kinase C (PKC) extracellular-regulated protein kinase (ERK) pathway[2], enhanced polyol pathway[3], oxidative stress[4], overproduction of advanced glycation end products[5], and enhanced growth factors and cytokines production[6].

The inappropriate activation of PKC has been implicated as a putative mediator in the pathogenesis of diabetic nephropathy based on both experimental animal models of type 1 diabetes and studies in cultured glomerular cells[7,8,9]. An increase in *de novo* synthesis of diacylglycerol (DAG) generated from glycolytic intermediates results in activation of PKC with induction of mesangial and glomerular dysfunction[10,11,12].

However, PKC is not a single entity, but consists of a family of at least 12 serine-threonine kinases with distinct expression patterns and cellular function[13]. These isoforms were first cloned in 1986 and have been divided on the basis of their regulatory domains into three larger subgroups: the classical PKC isoforms α, β I/II, and γ (regulated by calcium and DAG); the novel PKC isoforms δ, ε, η, and θ (regulated by DAG); and the atypical (noncalcium, non-DAG regulated) PKC isoforms ζ,, *ι,* /λ[14]. Specific PKC isoforms are activated by hyperglycemia, and the pattern of activation varies across tissues and cell types[15]. In addition, several PKC isoforms could be involved in diabetes-induced organ damage[16].

Evaluation of the PKC isoform activation pattern in diabetic nephropathy has gained great interest recently since specific PKC isoform inhibitors have been developed by several pharmaceutical companies[17]. In experimental diabetic nephropathy, PKC β inhibition has been related to the functional and histological abnormalities of glomeruli in two different diabetic animal models[10,18]. However, several investigators pointed to the high glucose-induced activation of novel and atypical PKC isoforms in the same animal models of diabetic nephropathy[19,20].

An emerging role for PKC isoforms in immune cell function has been identified recently[21]. Mice deficient in the conventional PKC β isoform were immunodeficient, exhibiting a loss of T-cell–independent antibody response[22]. On the other hand, mice deficient in PKC δ exhibited B-cell hyperactivation and autoimmunity[23]. Miyamoto et al.[24] suggested that PKC δ may regulate B-cell function by inhibiting interleukin 6 production. Similarly, PKC β appears to possess a similar ability to regulate B-cell function[21]. PKC β is activated on B-cell stimulation and functions in a negative feedback loop to inhibit further B-lymphocyte signaling cascade[21].

Data on renal PKC isoform expression in diabetic and nondiabetic glomerular diseases in humans are scarce, therefore, we conducted this study to evaluate the renal gene expression of classical PKC isoforms (α and β) in patients with diabetic nephropathy as well as patients with proliferative glomerulonephritis, including lupus nephritis (LN). We also examined the correlation between PKC gene expression and parameters of renal injury, including serum creatinine, estimated glomerular filtration rate (eGFR), and 24-h urinary protein excretion.

## PATIENTS AND METHODS

### Patients

Sixty outpatients were selected for the study between August 2005 and July 2006 based on renal biopsy findings. They were divided into three groups:

1. Twenty patients with type 2 diabetes and proteinuria — 11 males and nine females with mean age of 48.2 ± 12.9 years. Duration of diabetes ranged from 1.5 to 20 years (Mean 9.47 ± 6.36 years). Mean systolic blood pressure (SBP) was 141.87 ± 10.45 mmHg and mean diastolic blood pressure (DBP) was 87.45 ± 9.34 mmHg. Mean serum creatinine was 2.04 ± 0.85 mg/dl, mean eGFR using the Modification of Diet in Renal Disease equation[22] was 37.8 ± 17.8 ml/min/1.73 $m^2$, and 24-h urinary protein excretion rate was 3.61 ± 1.75 g. The main indications for renal biopsy were to exclude other causes of glomerular affection, lack of diabetic retinal disease and/or presence of microscopic hematuria, recent onset of heavy proteinuria, or shorter duration of diabetes. Renal biopsy showed either focal or diffuse diabetic glomerulosclerosis in the patients enrolled.
2. Twenty female patients with biopsy proven previously untreated focal or diffuse proliferative LN (class 3-4). Mean age was 24.7 ± 4.65 years, mean serum creatinine was 1.67 ± 1.51 mg/dl, mean eGFR was 69.6 ± 40.9 ml/min/1.73 $m^2$, and 24-h urinary protein excretion rate was 4.46 ± 5.01 g.
3. Twenty patients with biopsy proven previously untreated mesangioproliferative glomerulonephritis (MPGN) — 12 males and eight females. Mean age was 39.3 ± 14.59 years, mean serum creatinine was 3.32 ± 2.79 mg/dl, eGFR was 32.62 ± 29.56 ml/min/1.73 m2, and 24-h urinary protein rate was 3.32 ± 2.79 g.

None of the patients enrolled in this study was on angiotensin converting enzyme inhibitor/angiotensin receptor blocker therapy at the time of renal biopsy. Kidney biopsy samples were frozen at –80°C until analysis. The study design was approved by the local Research Committee and all patients gave consent for renal biopsy. Normal tissues from the normal pole of four kidneys removed because of renal tumor served as controls.

## Detection of PKC α and β Genes Expression by Semi-Quantitative Real-Time Reverse Transcriptase PCR (RT- PCR)

About 30 mg of kidney biopsy was homogenized, then centrifuged at 14,000 rpm for 10 min. The supernatant was examined for detection of protein kinase gene expression as follows:

1. RNA extraction: RNA was extracted from tissue homogenate by using SV-total RNA isolation system (Promega-Madison, USA) according to the manufacturer's protocol; the extracted RNA was measured by spectrophotometer at 280 nm.
2. RT-PCR: About 5 µg of RNA was reverse transcribed (RT) by using 12.5 µl of oligonucleotides primer denaturated at 70°C for 2 min. The denaturated RNA was placed on ice for 5 min. About 5 µl of RT buffer that contained 10 *M* Kcl, 50 m*M* tris Hcl, 0.5 m*M* dNTPS, and 200 units of moleny murine leukemia virus (MMLV) RT was used; the reaction conditions were 42°C for 1 h, followed by heating at 95°C for 5 min to stop the reaction. PCR reaction was performed by adding the PCR mix to about 5 µl of cDNA, the mixture contained 10 m*M*/l Hcl PH 8.3, 50 m*M* Kcl, 100 m*M* dNTPS, and 2.5 units of taq polymerase and about 10 µ*M* of each of sense and antisense primer of protein kinase α and β with the following sequence, respectively:

- Sense primer 5-CAGATGTCACTACAGCCTA-3-
     Antisense primer 5-TTCGGACTGTAACTGACGGT-3-
   - Sense primer 5-ATCTGCAAGTCACCGG-3-
     Antisense primer 5-GCCCATTGACCTAAAT-3-

The PCR cycling conditions were 95°C for 1 min for denaturation, followed by 55°C for 1 min for annealing, and 72°C for 45 sec; for 40 cycles with final extension at 72°C for 12 min.

3. Gel electrophoresis: 10 µl of PCR product was analyzed on 2% agarose gel with ethidium bromide staining and the product was visualized on ultraviolet transilluminator, then gel documentation was performed. PCR products were semi-quantitated by using a gel documentation system (Bio Doc Analyze) supplied by Biometra.

## Statistics

Data were presented as mean ± SD. Student's *t*-test was used to examine for statistically significant differences in PKC isoform gene expression between the examined groups. Pearson correlation coefficient was used to detect significant correlations between PKC gene concentrations and parameters of renal injury, including serum creatinine, eGFR, and 24-h urinary protein excretion. Results were considered significant for $p < 0.05$. Statistical analysis was performed using SPSS package version 11 (SPSS Inc., Chicago, IL, USA).

## RESULTS

The main demographic and laboratory parameters of the study group are shown in Table 1. LN patients were significantly younger and had significantly higher eGFR compared to diabetic patients with nephropathy ($p < 0.05$). Diabetic and MPGN patients, on the other hand, were comparable in age, gender distribution, renal function, and degree of proteinuria. Renal expression of PKC α and β genes were significantly increased in diabetic, LN, and MPGN patients compared to controls (Figs. 1 and 2).

**TABLE 1**
**The Main Demographic Characteristics, Laboratory Parameters, and PKC Isoform Expression Levels among the Study Groups**

| Parameters | DN | LN | MPGN |
|---|---|---|---|
| Age | 48 (12.9) | 24.7(4.65)** | 39.3(14.59) |
| Gender (M/F) | 11/9 | 0/20** | 12/8 |
| SBP (mmHg) | 141.87 ± 10.45 | 139.12 ± 15.65 | 129.54 ± 8.45 |
| DBP (mmHg) | 87.45 ± 9.34 | 86.97 ± 7.98 | 81.48 ± 8.75 |
| Serum creatinine (mg/dl) | 2.04 ± 0.85 | 1.67 ± 1.50 | 3.32 ± 2.79 |
| Blood urea (mg/dl) | 77.14 ± 14.67 | 60.62 ± 10.34 | 97.25 ± 22.45 |
| Serum albumin (g/dl) | 2.4 ± 0.92 | 2.2 ± 1.02 | 2.7 ± 0.87 |
| eGFR (ml/min) | 37.8 ± 17.8 | 69.6 ± 40.9** | 32.6 ± 29.5 |
| 24-h urinary protein/g | 3.61 ± 1.75 | 4.46 ± 5.01 | 3.32 ± 2.79 |
| PKC α gene (µg/ml) | 316.95 ± 152.94* | 185.97 ± 32.13 | 195.46 ± 46.45 |
| PKC β gene (µg/ml) | 18.20 ± 4.91 | 41.01 ± 14.03** | 39.93 ± 16.14** |

* $p < 0.05$ for diabetic nephropathy (DN) results compared to other two groups.

** $p < 0.05$ for the results of LN or MPGN groups compared to DN group.

Renal expression of the PKC α gene was significantly increased in diabetic patients with nephropathy compared to LN and MPGN patients (316.95 ± 152.94 µg/ml vs. 185.97 ± 32.13 and 195.46 ± 46.45 µg/ml, respectively, $p < 0.05$) (Figs. 1 and 3). On the other hand, renal PKC β gene expression was significantly increased in LN and MPGN compared to diabetic glomerulosclerosis (41.01 ± 14.03 and

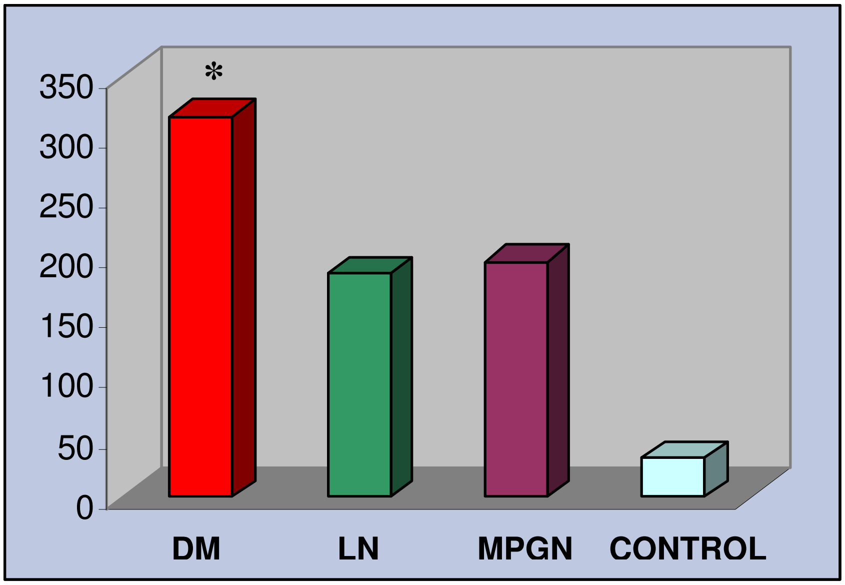

**FIGURE 1.** PKC α renal gene expression in the study groups (µg/ml). *$p < 0.005$ compared to MPGN and LN groups.

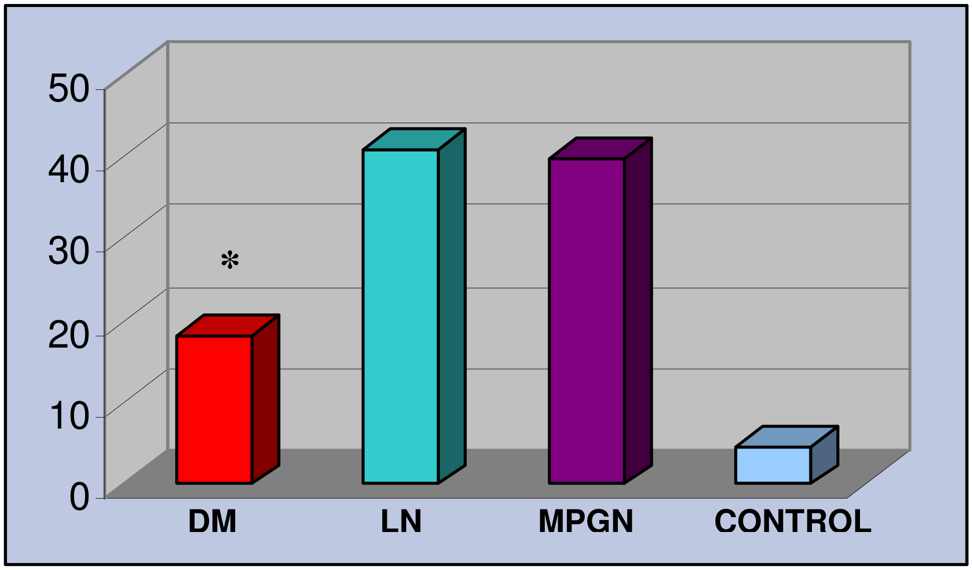

**FIGURE 2**. PKC β renal gene expression in the study groups (µg/ml). *$p < 0.005$ compared to LN and MPGN groups.

39.93 ± 16.41 µg/ml vs. 18.20 ± 4.91 µg/ml, $p < 0.05$) (Figs. 2 and 4). Renal PKC α and β gene expression in control tissues were 32.31 ± 0.36 and 4.67 ± 2.41 µg/ml, respectively.

There was significant positive correlation between renal PKC α gene concentrations and the degree of proteinuria in patients with diabetic nephropathy (Table 2, Fig. 5). Renal PKC β gene concentrations correlated significantly with age in the LN group (Table 2). No significant correlation was detected, however, between PKC isoforms α and β genes concentrations and serum creatinine and eGFR in the study groups.

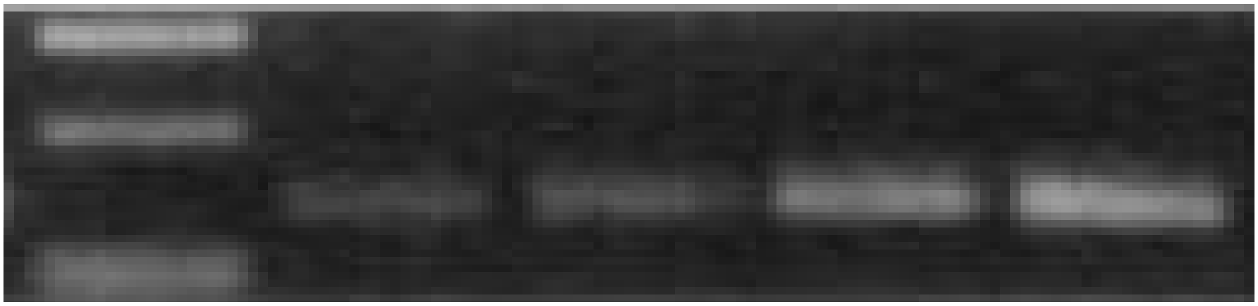

M LN MPGN DN DN

**FIGURE 3.** Ultraviolet transilluminated agarose gel electrophoresis shows PCR product of PKC α gene (104 bp) in different study groups (Lane M: PCR marker, Lane 2: LN case, Lane 3: MPGN case, Lanes 4 and 5: DN cases).

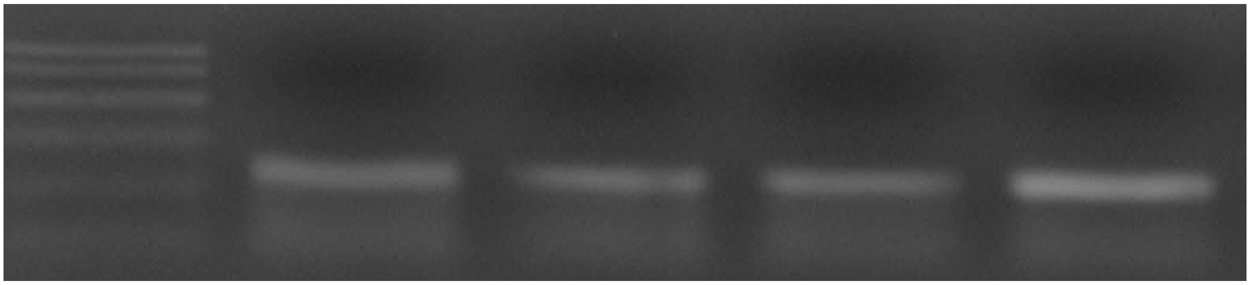

M MPGN DN DN LN

**FIGURE 4.** Ultraviolet transilluminated agarose gel electrophoresis shows PCR product of PKC β gene (254 bp) in different study groups (Lane M: PCR marker, Lane 2: MPGN case, Lanes 3 and 4: DN cases, Lane 5: LN case).

**TABLE 2**
**Correlation Analysis of Renal PKC α and β Levels and Baseline Patient Characteristics in the Three Study Groups**

| Parameters | DN<br>PKC α, PKC β | LN<br>PKC α, PKC β | MPGN<br>PKC α, PKC β |
|---|---|---|---|
| Age | R = –0.132, R = –0.474<br>P = 0.777, P = 0.282 | R = 0.684, **R = 0.902**<br>P = 0.62, **P = 0.002***  | R = –0.466, R = 0.288<br>P = 0.245, P = 0.489 |
| Serum creatinine | R = 0.087, R = 0.559<br>P = 0.852, P = 0.192 | R = –0.122, R = –0.472<br>P = 0.774, P = 0.238 | R = -0.601, R = 0.373<br>P = 0.115, P = 0.363 |
| Blood urea | R = –0.277, R = –0.015<br>P = 0.548, P = 0.975 | R = –0.075, R = –0.472<br>P = 0.861, P = 0.238 | R = 0.129, R = 0.279<br>P = 0.762, P = 0.503 |
| eGFR | R = –0.095, R = –0.407<br>P = 0.839, P = 0.364 | R = 0.235, R = 0.165<br>P = 0.576, P = 0.696 | R = –0.105, R = –0.111<br>P = 0.805, P = 0.794 |
| 24-h urinary protein | **R = 0.838,** R = 0.185<br>**P = 0.019*,** R = 0.693 | R = –0.581, R = –0.294<br>P = 0.131, P = 0.480 | R = 0.246, R = -0.232<br>P = 0.557, P = 0.580 |

* $p < 0.05$.

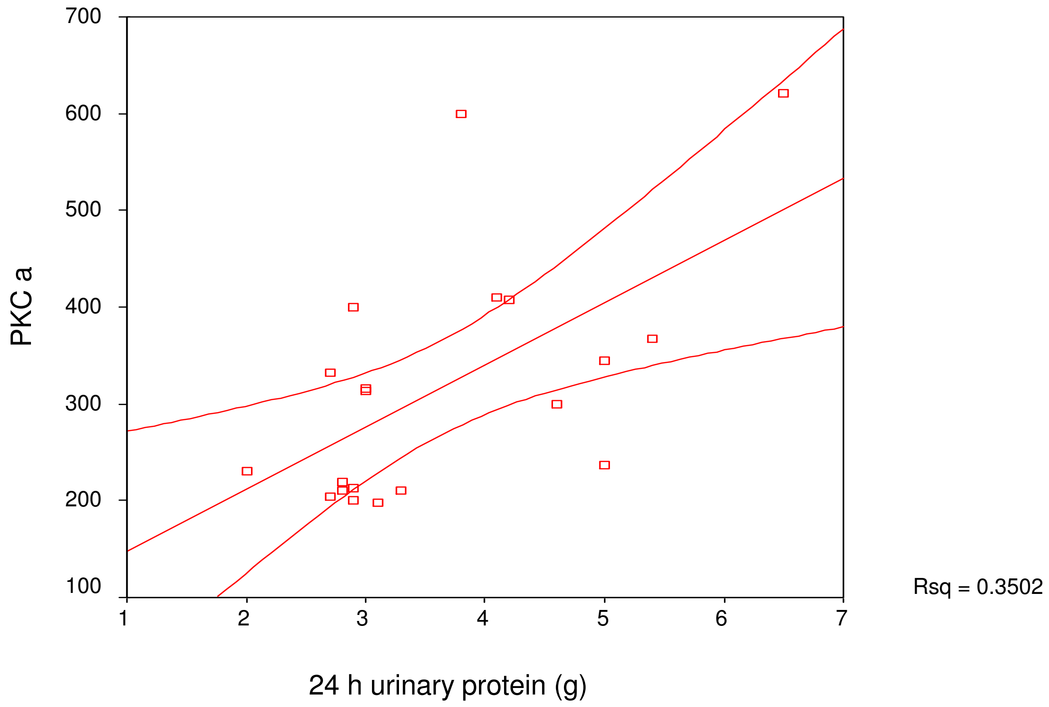

**FIGURE 5.** Correlation analysis between renal PKC α gene levels (μg/ml) and the 24-h urinary protein excretion rate (g/day) in patients with diabetic glomerulosclerosis.

## DISCUSSION

Two important findings were observed in the present study. First, we demonstrated enhanced renal gene expression of both PKC isoforms α and β in diabetic kidney tissues, LN, and MPGN, but in different patterns. PKC α gene expression was greatly increased compared to that of PKC β gene in diabetic kidneys. The ratio of renal PKC α/PKC β gene concentrations was 17.41 μg/ml in diabetic kidney tissues compared to 4.5 μg/ml in LN and 4.89 μg/ml in MPGN. Second, renal PKC β gene expression was significantly increased in LN and MPGN compared to diabetic nephropathy (41.01 ± 14.03 and 39.93 ± 16.14 μg/ml vs. 18.20 ± 4.91 μg/ml, respectively, $p < 0.05$). The enhanced PKC α renal gene expression in diabetic human kidney tissues detected in the present study is quite different from the predominant increase in PKC β activity in animal models of type 1 and 2 diabetes mellitus[10,18,25].

Experimental studies have demonstrated enhanced expression of PKC β in streptozotocin (STZ)-induced diabetic animals, a rodent model of type 1 diabetes, that was associated with a variety of glomerular and mesangial cell dysfunction, including mesangial matrix expansion and albuminuria[10]. Short-term treatment with orally administered PKC β inhibitor (LY333531) prevented early diabetes-induced glomerular dysfunction, including glomerular hyperfiltration, albuminuria, and enhanced mRNA expression of transforming growth factor $\beta_1$ (TGF-$\beta_1$) and extracellular matrix in STZ-diabetic rats[10]. Similarly, PKC β activity was 180% higher in db/db mice, a rodent model for type 2 diabetes, at 25 weeks of age compared to that observed in the nondiabetic db/m mice[18]. Administration of PKC β inhibitor reduced PKC activity to normal levels, normalized urinary albumin excretion rates, and ameliorated mesangial and extracellular matrix expansion and TGF-$\beta_1$ expression after 16 weeks of treatment[18]. PKC β inhibition attenuated macrophage recruitment and tubulointerstitial injury in Ren-2 diabetic rats[25].

On the other hand, other investigators reported enhanced renal expression of other PKC isoforms in diabetic kidney tissues in some animal models[19]. Immunoblotting of mesangial cellular fractions revealed increased membrane and nuclear expression levels of PKC α, δ, and ε after 48 h in 30 m*M*

glucose[19]. Kang et al.[26] demonstrated that PKC isoforms α and ε are activated in kidneys from STZ-induced diabetes rats. The case for PKC α isoform was strengthened by Kikkawa et al.[12] and Hempel et al.[27], who reported activation of PKC α in rat mesangial cells cultured under high glucose condition. In agreement with our results, *in vitro* experiments demonstrated that high glucose led to PKC α–dependent increase in TGF-$\beta_1$ expression in cultured vascular smooth muscle cells[28].

Recently, a specific role of individual PKC isoforms in the development of diabetic nephropathy was identified using knock-out (KO) mice[29,30,31,32]. The PKC α isoform seems to be an important mediator of glucose-induced glomerular changes, including increase in vascular endothelial growth factor (VEGF) and its receptor, loss of heparan sulfate proteoglycans (the carrier of negative charges in the basal membrane), and development of albuminuria[29,30]. The PKC β isoform, on the other hand, seems to be included in the activation of the profibrotic pathway through increased expression of TGF-$\beta_1$ and connective tissue growth factor (CTGF)[31,32]. Thus, the coexpression of these two isoforms in the diabetic kidney tissues observed in the current study explained the advanced glomerular structural and functional changes in this group of diabetic patients, including albuminuria, glomerular sclerosis, and renal insufficiency. In addition, PKC α gene expression correlated positively with urinary protein excretion in diabetic patients in the current study, and that further supports its role in the development of albuminuria in this group of patients.

The renal coexpression of both PKC α and β genes, with predominant α isoform expression, in diabetic nephropathy might explain the inconsistent effects of selective PKC β inhibition in type 2 diabetic patients with nephropathy[33,34]. Treatment with ruboxistaurin (selective PKC β inhibitor) reduced albuminuria and maintained eGFR over 1 year in type 2 diabetic patients with nephropathy enrolled in a pilot study that included 123 patients[33]. Later on, a larger study that enrolled 1157 diabetic patients (type 1 and 2) with diabetic retinopathy treated with ruboxistaurin did not confirm a potential benefit from selective PKC β on kidney outcomes, including doubling of serum creatinine, development of advanced kidney disease (stage 4 to 5), and death[34].

A novel finding in the present study was to demonstrate an increased renal expression of PKC β gene in MPGN and LN patients compared to the diabetic nephropathy group. It was also noted that the PKC α gene was also coexpressed in LN and MPGN, but to a lesser extent in comparison with diabetic kidney tissues. Taken together, these data suggest a major role for PKC isoforms α and β in the pathogenesis of proliferative glomerulonephritis, including LN. This also may suggest a potential therapeutic role for PKC inhibition in the treatment of proliferative glomerulonephritis.

Data on renal activation of the PKC pathway in glomerulonephritis in humans are scarce. For instance, the PKC δ isoform was shown to mediate TGF-$\beta_1$–induced collagen I expression in human mesangial cells[35]. Inhibition of PKC δ decreased basal- and TGF-$\beta_1$–stimulated collagen I production and mRNA expression[35]. PKC pathway activation was demonstrated to follow complement activation and assembly of C5b-9 on glomerular epithelial cells in experimental membranous nephropathy[36]. Enhanced expression of PKC isoforms α and β was detected in renal biopsy tissues of patients with membranous nephropathy in parallel with C5B-9 activation[37]. Certain PKC isoforms may have immunomodulatory functions as enhanced PKC isoform β expression protects macrophages from nitric oxide–induced apoptosis[38].

Similarly, little is known regarding functions of PKC isoforms in the pathogenesis of LN. Some emerging data suggest a role for PKC β in B-lymphocyte function regulation[21]. PKC β is activated on B-cell receptor stimulation, in response to increased concentrations of DAG and $Ca^{2+}$[21]. PKC β has a definite role in mediating positive effects downstream of Bruton's tyrosine kinase (Btk). PKC β can also inhibit Btk through a negative feedback loop[39].

To conclude, we have shown a distinct pattern of PKC α and β gene activation in diabetic nephropathy, LN, and MPGN in humans that differs from that observed in experimental studies. Future studies are needed to study the potential therapeutic application of specific PKC α and β inhibitors in the treatment of diabetic- and immune-mediated proliferative glomerular diseases.
